# Fast Conversion of Ionic Liquids and Poly(Ionic Liquid)s into Porous Nitrogen-Doped Carbons in Air

**DOI:** 10.3390/ijms17040532

**Published:** 2016-04-08

**Authors:** Yongjun Men, Martina Ambrogi, Baohang Han, Jiayin Yuan

**Affiliations:** 1 Department of Colloid Chemistry, Max Planck Institute of Colloids and Interfaces, Potsdam D–14476, Germany; yongjun.men@gmail.com (Y.M.); martina.ambrogi@mpikg.mpg.de (M.A.); 2 National Center for Nanoscience and Technology, Beijing 100190, China; hanbh@nanoctr.cn

**Keywords:** ionic liquid, poly(ionic liquid)s, porous carbon

## Abstract

Ionic liquids and poly(ionic liquid)s have been successfully converted into nitrogen-doped porous carbons with tunable surface area up to 1200 m^2^/g at high temperatures in air. Compared to conventional carbonization process conducted under inert gas to produce nitrogen-doped carbons, the new production method was completed in a rather shorter time without noble gas protection.

## 1. Introduction

Carbonization is typically a high-temperature reaction converting organic substances into carbon-rich products through pyrolysis or destructive distillation [1,2,3,4,5,6,7,8,9,10,11,12,13,14,15,16,17,18,19]. This process is ideally (though not necessarily) conducted under inert atmosphere, such as N_2_ or Ar, because air at high temperature may quickly oxidize the carbons formed *in situ* into gaseous compounds, like CO or CO_2_, leading to degradation of the carbon products. Particularly challenging is producing porous carbons in air, as the amplified surface contact accelerates the aerobic degradation. It is therefore a matter of scientific curiosity whether one can produce porous carbons in the presence of air. Stucky *et al.* [20] recently reported the production of hollow, porous carbon spheres, which relied on physical confinement by a dense TiO_2_ barrier shell to block the access of O_2_. In fact, physcial confinement has been applied in industrial carbonization processes carried out in sealed chambers, where oxygen is first quickly consumed by the carbon precursors, and then it becomes a process protected by the remaining N_2_. Strictly speaking, carbonization under confinement is not a truly aerobic carbonization process, as it eliminates oxygen and produces a nitrogen-rich environment to protect the carbonization process. Our interest here is to discuss the production of porous carbons with the presence of air throughout the carbonization process.

Ionic liquids (ILs) are ionic materials in a liquid state below 100 °C. Poly(IL)s (PILs) incorporate ILs as their repeating units, and show improved mechanical stability and processability compared to ILs [21,22,23,24,25]. Recently, both ILs and PILs, due to their high nitrogen content and thermostability, have been used as precursors to nitrogen-doped carbons under N_2_ or in a confined environment [21,26,27,28,29,30,31,32]. It is known that nitrogen doping is able to adjust the physical properties of carbon via increasing the overall electron density at the Fermi level, which improves the electric conductivity and oxidation stability. Our previous research havs demonstrated the fire-retardant properties of these porous carbons [33]. These properties allow these carbons to remain stable and preservable in air for a defined time at high temperatures without severe mass loss. Bearing this in mind, herein we aimed to introduce a truly aerobic synthetic route to rapidly produce porous nitrogen-doped carbons from IL and PIL at elevated temperatures without the assistance of inert gas or confinement [34]. This method was further coupled with the superior processability of PILs to activate, biomass, hereby exemplified with nature cotton into porous carbon foam in air.

## 2. Results and Discussion

### 2.1. Carbonizaiton of Ionic Liquid in Air

**Scheme 1:**
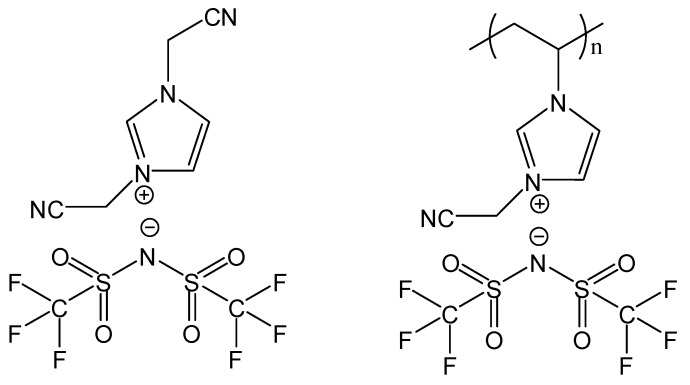
Chemical structures of the ionic liquid (**left**) and poly(ionic liquid) (**right**) used in this work for aerobic carbonization.

**Figure 1:**
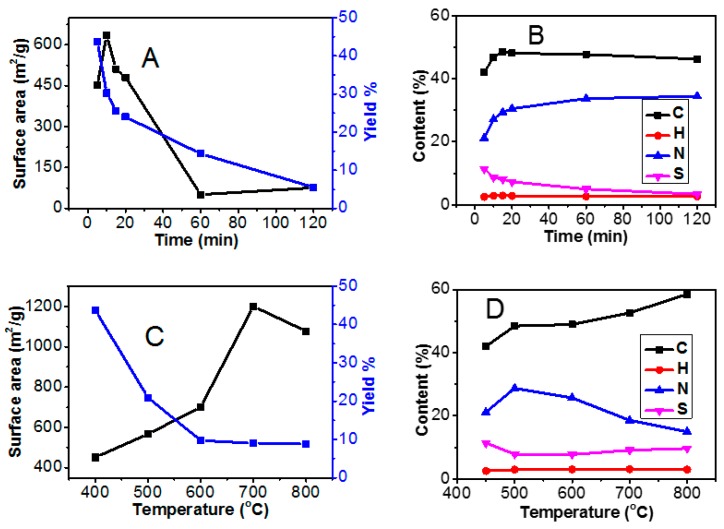
Plots of (**A**) carbonization yield and *S*_BET_ (Brunauer–Emmett–Teller specific surface area) *vs.* the pyrolysis time (Entries 1–6 in Table 1); (**B**) the C/H/N/S content of carbon products prepared at 450 °C at different time; (**C**) carbonization yield and *S*_BET_ *vs.* the pyrolysis temperature (Entries 7–10 in Table 1); (**D**) the C/H/N/S content of carbon products prepared via 5 min pyrolysis at different temperatures.

In an initial test, the model IL (Scheme 1) was fired in air at 1300 °C with a butane/propane gas burner. It was observed that the IL turned from a transparent liquid to a turbid-black solution and rapidly to a dark solid in *ca.* 20 s. The dark residue was further combusted totally after 2 min. As this process was rather quick, it is impossible to stop at a specific stage to isolate the carbon product. To slow down this process, we lowered the pyrolysis temperature stepwise. It was found that at 450 °C, even after 1 h, a bulk black residue remained. With this positive observation, we performed more quantitative tests at this temperature. The same IL placed in different crucibles was then transferred into an air oven pre-heated to 450 °C. The samples were taken out sequentially after 5, 10, 15, 30, 60, and 120 min, respectively. All samples were black and used for yield determination, elemental analysis and gas sorption measurements. The results are summarized in Table 1 and Figure 1.

As can be seen from Entries 1–6 in Table 1 and Figure 1A, the carbonization yield drops gradually along extended holding time at 450 °C, from 43.7 wt % for 5 min to only 5.6 wt % for 120 min. This trend is actually expected, as long-term exposure to air at 450 °C will, though slowly, degrade the carbon products, because the favorable nitrogen doping retards but does not stop the oxidation process. When the dwelling time is limited to 60 min, the pore sizes are mainly less than 2 nm (Figure S1), *i.e.*, microporous. The N_2_ sorption measurements show a peak value of *S*_BET_ of 635 m^2^/g after exactly 10 min (Entry 2 in Table 1). Samples prepared either earlier or later than this carry lower *S*_BET_ values. In our opinion, this implies a complex effect from two competitive reactions, namely the pore formation reaction and the oxidative etching of carbons. The pore formation involves the trimerisation of the cyano groups to build up a thermally stable network and the subsequent decomposition of the bis(trifluoromethanesulfonyl)imide (Tf_2_N) anion to introduce the pores. The decomposition of Tf_2_N anion at 450 °C can be monitored by elemental analysis, *i.e.*, the residue sulfur in the carbon products, since in the IL only Tf_2_N contains sulfur. Figure 1B shows a plot of the C/H/N/S content *vs.* the carbonization time. A quick decrease in sulfur content occurs in the first 15 min, followed by a relatively smooth plateau close to zero. This result proves the pore indeed formed via anion decomposition, dominantly, in the initial 15 min. It is also supported by the rapid weight loss (yield) in the same period (Figure 1A), as Tf_2_N accounts for 66 wt % of the IL. The oxidative etching of the *in situ* generated carbon proceeds as long as the sample remains in air at 450 °C. It burnt away the formed porous carbon, lowering the *S*_BET_ and the yield gradually. It is thus not surprising that the highest *S*_BET_ value peaks at 10 min, before which the pore formation process prevails over the porous carbon degradation. This is a rather short period compared to the hours typically taken by the usual carbonization process under noble gas protection, not to mention the initial time for replacement of air by inert gas in the oven.

Carbonization temperature is another effect studied here by fixing the holding time to 5 min for all samples. The results (Entries 7–10 in Table 1) are displayed in Figure 1C. Generally, at high carbonization temperatures, a lower yield and more rapid weight loss were observed. For instance, above 600 °C the carbonization yield is less than 10 wt %, while at 450 °C it is as high as 43.7 wt %. The *S*_BET_, however, increases with the carbonization temperature. A maximum *S*_BET_ of 1200 m^2^/g was found at 700 °C, with a pore size less than 4 nm (Figure S2). Nevertheless, at this temperature the carbonization yield is undesirably low, only 9 wt %. The elemental analysis in Figure 1D shows that the carbon content increases with the temperature due to the accelerated decomposition of the carbon-poor anion and the enhanced elimination of nitrogen and hydrogen atoms at high temperatures.

**Figure 2:**
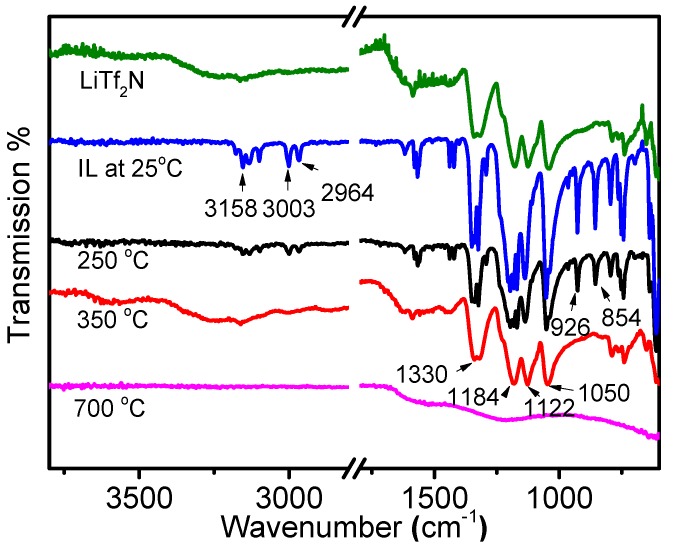
FTIR spectra of carbonization products of IL prepared after 5 min at temperatures from 25 to 700 °C.

To get greater insight into thermal decomposition in air, Fourier transform infrared (FTIR) spectrometry measurement was performed on carbons prepared after 5 min of pyrolysis at different temperatures. The spectra are displayed in Figure 2. At 250 °C, the spectrum pattern of IL can be well indexed to each band. No obvious change was found when compared to that at room temperature. Right from 350 °C, the FTIR bands of the imidazolium cation structure at 854 and 926 cm^−1^ (=C–H bending from imidazole ring) vanished, indicating the thermal structure reorganization occurred and the fire retardant structure formed. The Tf_2_N anion bands (1050, 1122, 1184 and 1330 cm^−1^) remain untouched at 350 °C due to its superior thermal stability. Nitrogen sorption measurement of the carbon sample at 350 °C indicates limited surface area (<10 m^2^/g), since, as mentioned above, the pores form only after the anion decomposition. A large *S*_BET_ was received once the oven temperature surpassed 350 °C to decompose the Tf_2_N anion, for example 1200 m^2^/g at 700 °C. FTIR spectrum of this sample at 700 °C in Figure 2 shows that the Tf_2_N anion-related bands completely disappeared. This result is understandable, since the surface area comes from the pores left by Tf_2_N.

**Figure 3:**
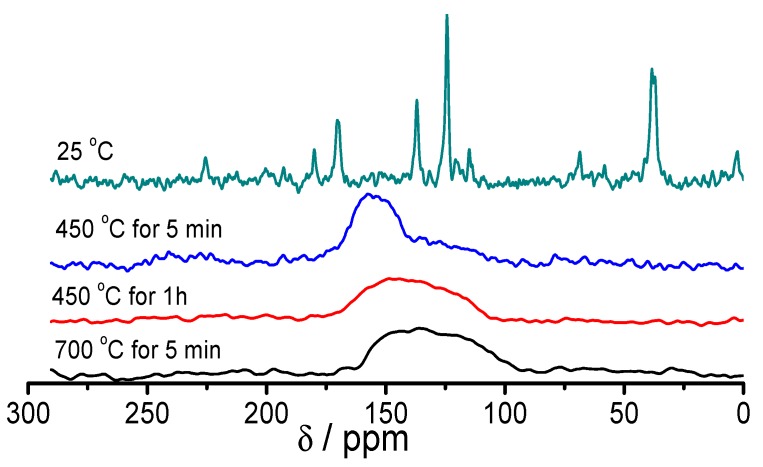
Solid state ^13^C-NMR spectra of IL prepared at different holding time and temperatures.

The atomic structure of the carbon products was investigated by solid state ^13^C-NMR measurements [35]. The recorded spectra are displayed in Figure 3. At 450 °C for 5 min, the original sharp peaks from the IL at room temperature were replaced by two broad overlapping bands, *i.e.*, the aromatic carbons (100–140 ppm) and the isolated melon structure (140–170 ppm) [36]. This indicates the occurrence of the trimerisation reaction of the nitrile groups in the IL. No sharp peak at 50–80 ppm was observed so that epoxy or ether structures were absent in the carbon matrix. Annealing at 450 °C for 1 h promotes the transition of the isolated melon structure into aromatic carbons, indicated by the dramatic shrinkage of the melon band between 140 and 170 ppm. When the sample was processed at 700 °C for 5 min, a similar pattern was observed. It implies either extending the annealing period (at 450 °C from 5 min to 1 h) or enhancing the temperature (from 450 to 700 °C, keep the holding time for 5 min) leads to the promoted formation of aromatic carbon frameworks.

### 2.2. Carbonizaiton of Poly(Ionic Liquid) and Poly(Ionic Liquid) Coated Cotton in Air

All aforementioned analysis indicates that the formation of nitrogen-doped porous carbons directly from pyrolyzing IL in air without any physical confinement is feasible. To extend this method to other materials, the first one we tried is the PILs—that is, polymers incorporate ILs as their repeating units, with similar structure (here, the one shown in Scheme 1) [22,23,24,25,30,31,37]. As shown in Table 2 (Entries 11–15), a carbonization yield of 30.4 wt %, a high nitrogen content of 19 wt % and a *S*_BET_~749 m^2^/g for PIL were obtained at 450 °C after 10 min (Entry 13). A reduced holding time at 450 °C, such as 5 or 6 min drops *S*_BET_ below 200 m^2^/g (Entries 11 and 12), and pore sizes are majorly between 2.5 and 4 nm (Figure S3), *i.e.*, small mesopores. At higher temperatures, such as 700 and 800 °C, the *S*_BET_ increases further to 879 and 955 m^2^/g, respectively (Entries 14 and 15), and the micropores turned dominant. These experiments prove that the generation of nitrogen-doped porous carbon in air via the IL and PIL route is practically applicable by carefully choosing the pyrolyzing temperature and holding time.

### 2.3. Carbonizaiton of Poly(Ionic Liquid) Coated Cotton in Air

**Figure 4:**
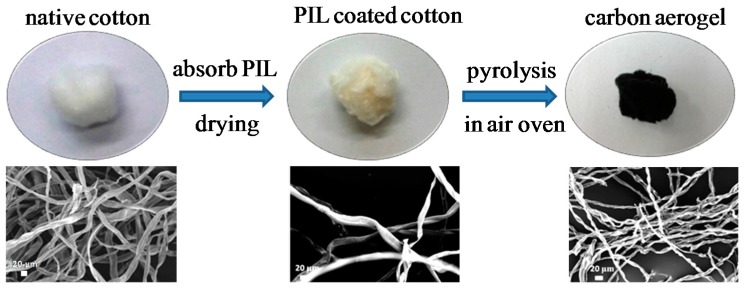
Preparation of carbon foam via carbonization in air and their corresponding SEM images (scale bar: 20 µM).

A further description of this method is by carbonization of PIL-coated cotton (PIL-cotton) in air. The design strategy is that the PIL, as demonstrated, can form a fire-retardant carbon network at around 350 °C, which can be utilized as a protection layer on other non-fire-retardant carbon precursors based on the excellent processibility and mechanical stability of PILs. Here, cotton was selected as the non-fire-retardant precursor due to its low cost and easy shaping properties. As shown in Figure 4, PIL could coat cotton fibers by soaking cotton in a PIL solution in acetonitrile (10 wt %) followed by drying in air. Due to the large pore volume of nature cotton, it is possible to test the aerobic production of porous carbon aerogel from biomass. When placed in air at 450 °C, pure cotton was rapidly burnt away in few seconds, while the PIL-cotton was ignited first, self-quenched within a few seconds and ended up with a dark shrunk foam (Figure 4), which is deformable. Table 2 (Entries 16–22) summarizes the experiments under different PIL loading content and dwelling time. All PIL-cotton carbon aerogels possess a S_BET_ value above 400 m^2^/g and micropores (Figure S4). Even when only containing 5 wt % of PIL, the S_BET_ value reached to 410 m^2^/g. The reason might be that the porous fire retardant protecting layer is not dense and thick enough to isolated most of the oxygen from air, and the leaking oxygen can etch the formed carbons as activating agent to increase the volume of micropores and decrease the yield. Compared to pure PIL in entries 11–15, the carbonization yield for carbon aerogels is lower, less than 20 wt % in general. This is understandable, since pure cotton under the same condition leaves nothing. The SEM characterization in Figure 4 proves the good preservation of the microstructure of cotton fiber morphology into the carbon foam. This explains the favorable shape deformability of the carbon foam.

## 3. Materials and Methods

### 3.1. Materials

1-Vinylimidazole (99%), bromoacetonitrile (97%), 1,3-bis(cyanomethyl)imidazolium bis(trifluoromethylsulfonyl)imide (≥94%), lithium bis(trifluoro methanesulfonyl)imide (99.95%) were obtained from Sigma-Aldrich (St. Louis, MO, USA) and used without further purification. 2,2′-Azobis(2-methylpropionitrile) (98%, Sigma-Aldrich) was recrystallized from methanol. The solvents and other materials were used as received.

### 3.2. Synthesis of the PIL Poly(3-cyanomethyl-1-vinyl imidazolium TFSI)

For the IL monomer synthesis, 0.1 mol of 1-vinylimidazole was dropwise added into a bromoacetonitrile (0.1 mol) solution in diethyl ether (50 mL) in a round bottom flask equipped with a condenser and a magnetic stirrer at 25 °C. After overnight stirring, a white precipitate appeared and was isolated. It was washed with ether (50 mL) 3 times and dried at room temperature under high vacuum overnight till constant weight. From this dried monomer powder, 8.4 g of the synthesized monomer was mixed with 160 mg of 2,2′-azobisisobutyronitrile (AIBN) in 100 mL of DMSO in a 250 mL Schlenk glass. After degassing by high vacuum and back-filling with argon, the polymerization was conducted at 90 °C for 12 h. A light yellow solid was obtained by precipitating the mixture solution twice in acetone and dried at 60 °C under vacuum to a constant weight. Yield: 80%. ^1^H-NMR (DMSO-*d*_6_, λ, ppm): 8.9–9.6 (1H), 7.0–8.5 (2H), 5.2–5.7 (2H), 3.5–4.6 (1H), 2.0–2.8 (2H). The apparent molecular mass and the polydispersity index of the PIL polymer was measured by gel permeation chromatography to be 1.15 × 10^5^ g/mol and 2.95, respectively, using polystyrene as standard.

### 3.3. Carbonizaztion of IL/PIL in Air

An air oven was set at a desired temperature, and then the crucibles with PIL or IL samples were transferred inside directly for carbonization. After the pyrolysis, the crucible was taken out of the oven using tweezers and cooled down to room temperature.

### 3.4. Characterization Methods

Nitrogen sorption experiments were performed on a Quantachrome Autosorb-1 or Quadrasorb at liquid nitrogen temperature, and data analysis was performed with Quantachrome software. The surface area was calculated using the Brunauer–Emmett–Teller (BET) equation. The pore size distribution was calculated using the density functional theory (DFT) method. The samples were degassed at 200 °C for 15 h before measurements. Elemental analysis was performed for carbon, hydrogen, sulphur and nitrogen using a Vario EL Elementar.

## 4. Conclusions

In conclusion, we discussed and demonstrated the feasibility of producing nitrogen-doped porous carbons straightforwardly in air without protection gas or confinement by using IL/PIL here as an example. The success of this unique production mode is attributed to the high nitrogen content in the IL/PIL cation and a self-templating mechanism due to the large-sized anion. Compared to the conventional carbonization process under noble gas protection, this aerobic carbonization method is more rapid. Based on this prototype mode of the air-carbon synthesis, it is of future interest to increase the carbonization yield while keeping the high-surface-area nature of the carbon products during the synthesis. Additionally, the applicability of this synthetic strategy can be further expanded by employment of other cheaper and greener carbon precursors. For example, various fire retardant materials can be applied after careful design to generate *in situ* porous hybrid carbon materials by mixing with suitable dissipative templates without noble gas protection.

## Figures and Tables

**Table 1:** Analysis results of carbon products obtained via pyrolysing ionic liquid (IL) in air at different holding times and temperatures. (*S*_BET_: Brunauer–Emmett–Teller specific surface area.)

Entry	Temperature (°C)	Time (min)	*S*_BET_ (m^2^/g)	Yield (wt %)	Nitrogen Content (wt %)
1	450	5	451	43.7	21.2
2	450	10	635	30.3	27.1
3	450	15	511	25.6	29.4
4	450	20	481	24.0	30.5
5	450	60	51	14.4	33.7
6	450	120	76	5.6	34.4
7	500	5	567	21.0	28.2
8	600	5	701	9.8	25.7
9	700	5	1200	9.0	18.6
10	800	5	1077	8.8	15.0

**Table 2:** Analysis results of carbon products obtained via pyrolysing poly(IL)s (PILs) (Entries 11–15) and cotton-PIL composites (Entries 16–22) in air at different holding times and temperatures.

Entry	Material	Temperature (°C)	Time (min)	*S*_BET_ (m^2^/g)	Yield (wt %)	N (wt %)
11	PIL	450	5	33	57.9	13.2
12	PIL	450	6	174	48.1	14.8
13	PIL	450	10	749	30.4	19.0
14	PIL	700	5	879	13.5	13.6
15	PIL	800	5	955	11.8	8.8
16	cotton-PIL (0) ^[a]^	450	5	-	0	0
17	cotton-PIL (5)	450	5	410	3.8	6.9
18	cotton-PIL (10)	450	5	575	9.6	8.8
19	cotton-PIL (15)	450	5	595	12.6	11.0
20	cotton-PIL (15)	450	10	517	6.5	13.3
21	cotton-PIL (30)	450	5	719	17.6	13.1
22	cotton-PIL (30)	450	10	525	10.3	12.9

^[a]^ The number in bracket in column “material” means the weight content of PIL in the material.

## Supplementary Materials

Supplementary materials can be found at http://www.mdpi.com/1422-0067/17/4/532/s1.
