# Supplementary material for: Fast Conversion of Ionic Liquids and Poly(Ionic Liquid)s into Porous Nitrogen-Doped Carbons in Air
Source: Int J Mol Sci. 2016 Apr 8;17(4):532. doi: 10.3390/ijms17040532 (PMC4848988; doi:10.3390/ijms17040532)
Supplement: Supplementary file 1 [file ijms-17-00532-s001.pdf]

Supplementary Materials: Fast Conversion of Ionic Liquids and Poly(Ionic Liquid)s into Porous Nitrogen-Doped Carbons in Air

Yongjun Men, Martina Ambrogi, Baohang Han and Jiayin Yuan

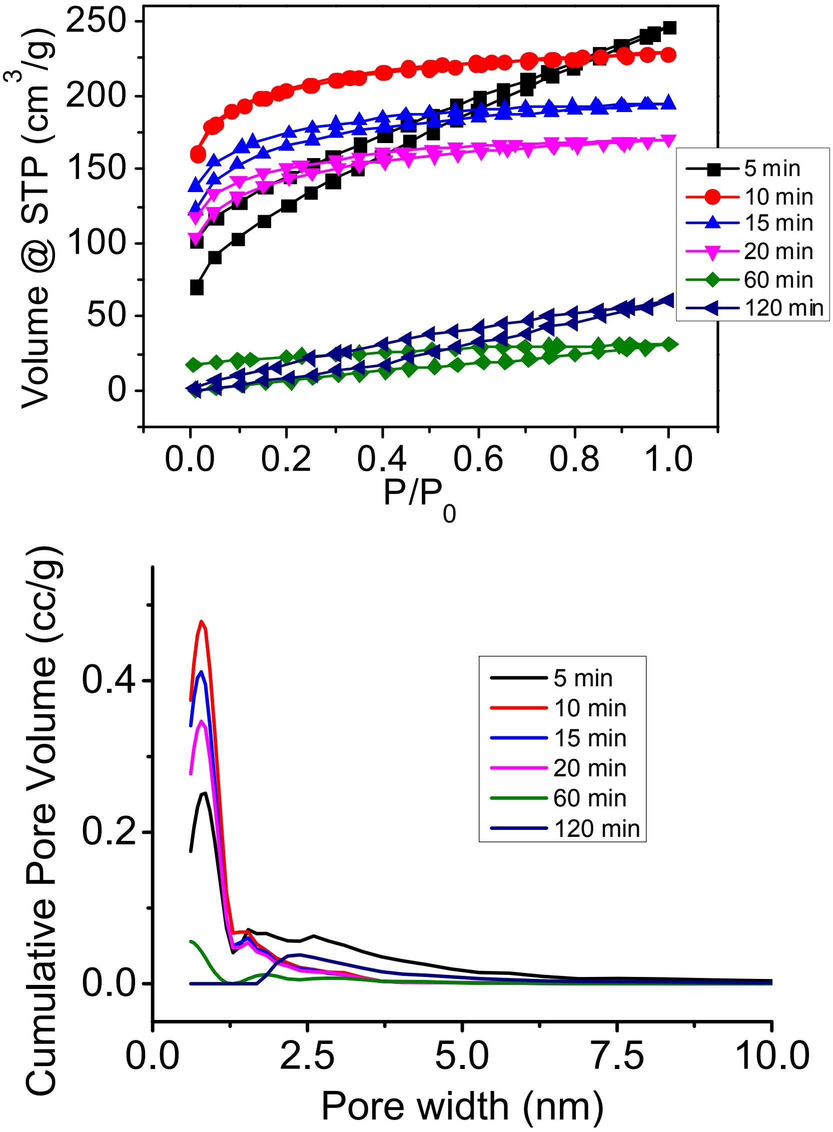

Figure S1. Nitrogen sorption isotherms and pore size distributions of carbonaceous products (entries 1–6 in Table 1) obtained at 450 °C from ionic liquid (IL) with different holding times.

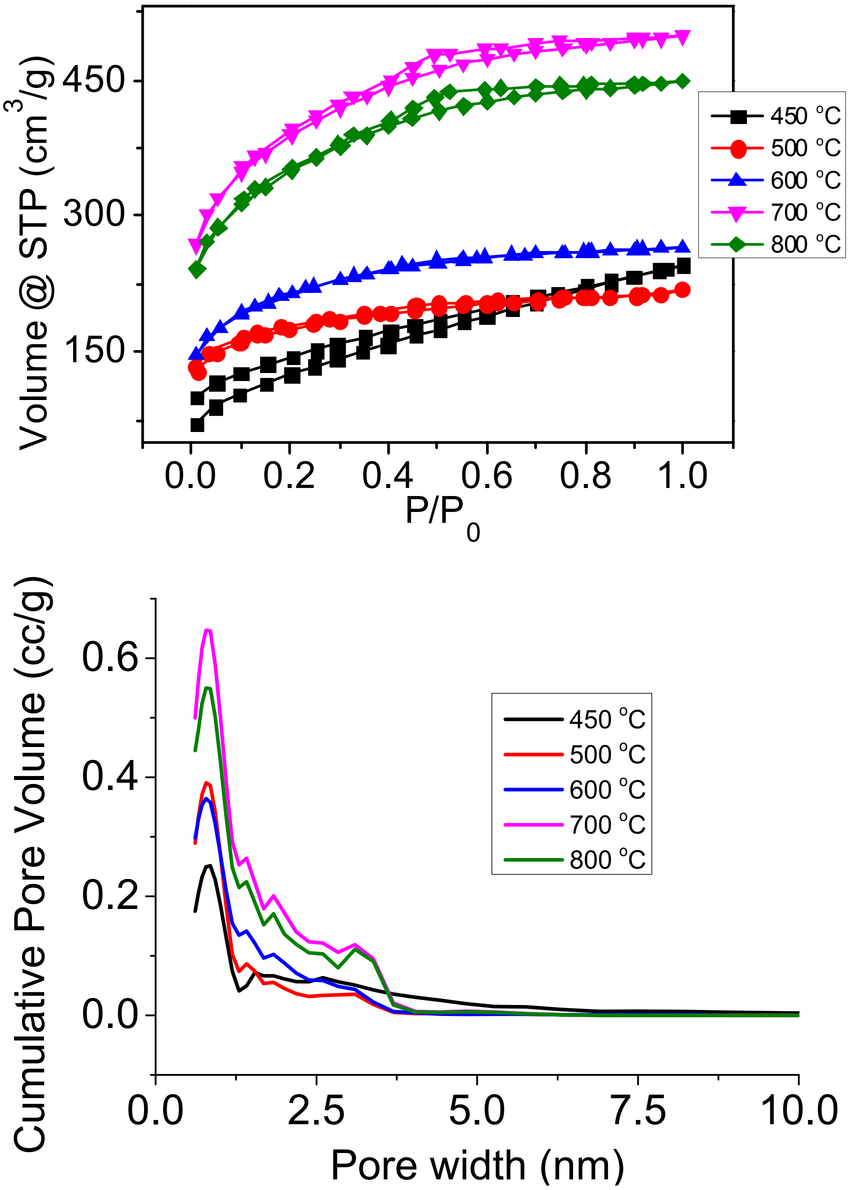

Figure S2. Nitrogen sorption isotherms and pore size distributions of carbonaceous products (entries 1, 7–9 in Table 1) obtained at different carbonization temperatures from IL with a holding time at 5 min.

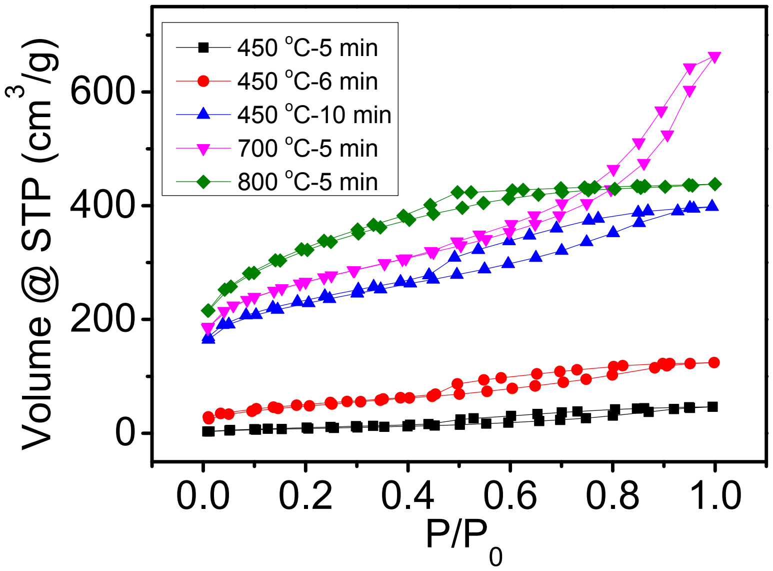

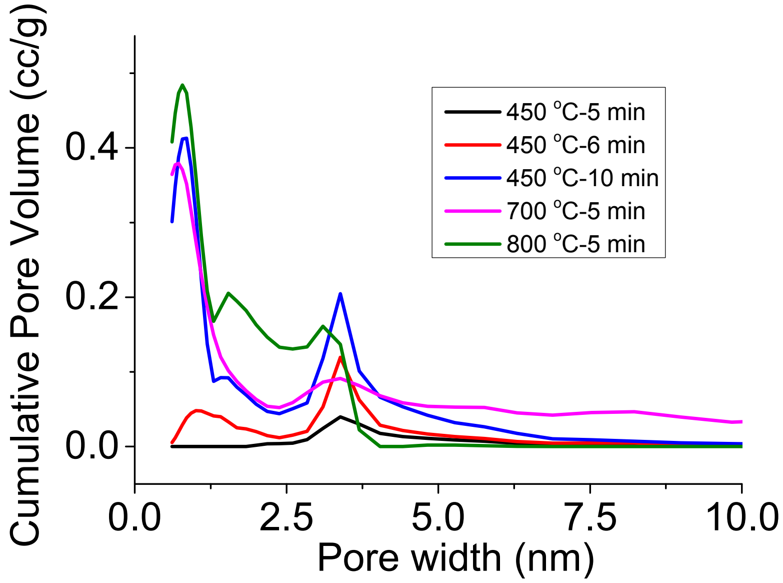

Figure S3. Nitrogen sorption isotherms and pore size distributions of carbonaceous products (entries 11–15 in Table 1) obtained at different carbonization temperatures from poly(IL) (PIL) with different holding times.

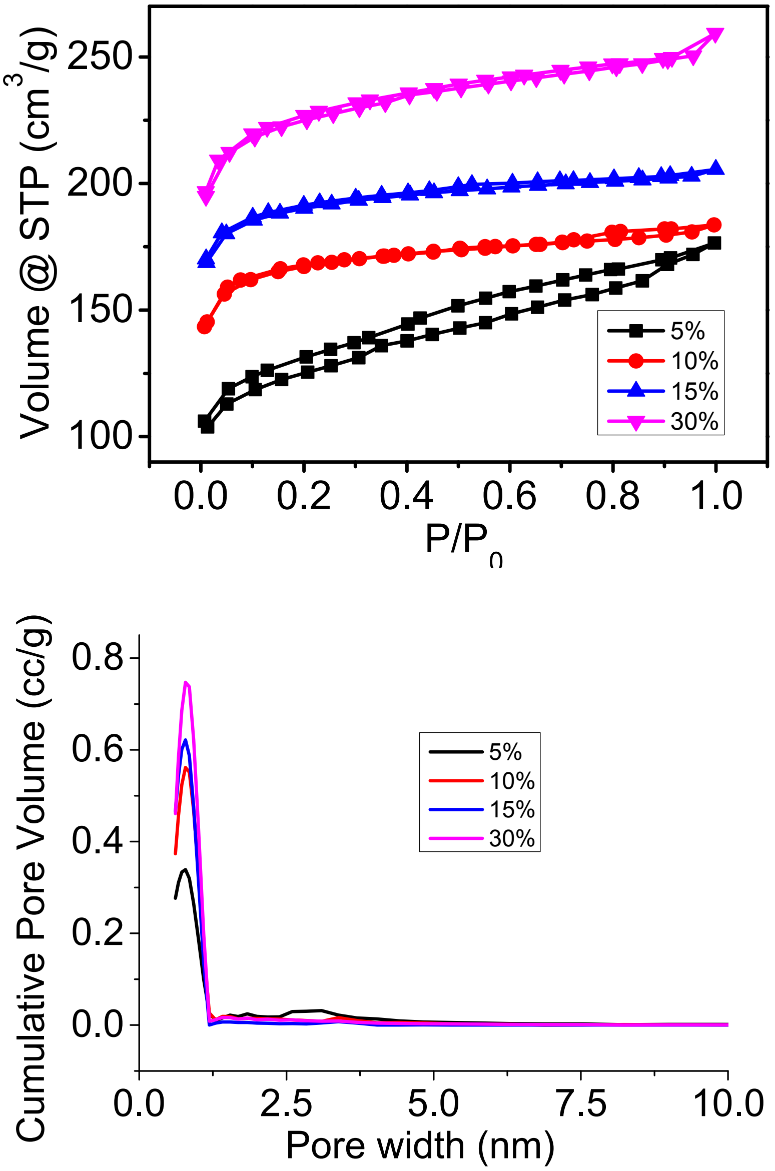

Figure S4. Nitrogen sorption isotherms and pore size distributions of carbonaceous products (entries 17–19 and 21 in Table S1) obtained at 450 °C for 5 min from different weight fractions of PIL in the PIL-cotton hybrids.
